# Immigrant women’s experiences with Norwegian maternal health services: implications for policy and practice

**DOI:** 10.1080/17482631.2022.2066256

**Published:** 2022-04-17

**Authors:** Lydia Mehrara, Trude Karine Olaug Gjernes, Susan Young

**Affiliations:** a Faculty of Social Sciences, Nord University, Bodø, Norway; b Social Work and Social Policy, The University of Western Australia, Crawley, WA, Australia

**Keywords:** Childbirth, immigrant women, maternal health, medicalization, Norway, pregnancy, universalism

## Abstract

**Purpose:**

Navigating a health system which differs considerably from one’s own can be a challenging process. Navigating this in seeking maternal health care can be additionally daunting. This article explores how immigrant women from diverse countries and ethnic backgrounds experienced and navigated the Norwegian maternal health service during pregnancy and childbirth.

**Method:**

Eleven semi-structured interviews collected between 2019 to 2020 and analysed thematically informed this analysis.

**Findings:**

Principles of universalism underpinning all social and health policy in Norway, expect equality of service provision and access. These principles result in less individual choice. The women in this study found these contrary to their expectations of care but responded differently, with some experiencing the health provision as liberating while others distrusted that they were receiving the best care. A focus of concern was the expectation of more medicalized services. While some of these women used their own resources to circumvent the Norwegian health provisions, the implications for the health system extend beyond these women’s experiences.

**Conclusions:**

The analysis suggests a need to encourage those whose expectations of care differ to build trust in institutions providing care. This article contributes to knowledge on the implications of diversity on Norway’s universal health system.

## Introduction

This article explores the experiences of immigrant women from diverse cultural backgrounds with the Norwegian maternity health care during their pregnancies and births. The women immigrated to Norway for various reasons from countries including China, Germany, Indonesia, India, Israel, Iran, and Serbia. In the interests of ensuring promised anonymity, no further details about the background of the participants are provided.

When mothers encounter a new maternity care system that is culturally and organizationally different from what they are used to, it may affect their interaction with the health professionals and result in undesirable experiences (Ladha et al., 2018, p. 66). Studies from Sweden and Finland, which have health care systems similar to Norway, indicate that immigrant women, although a heterogenous group, are more vulnerable to the risk of adverse pregnancy outcomes and poorer experiences with the maternity care services because of cultural sensitivity or communication issues compared to native-born women (Degni et al., 2014; Råssjö et al., 2013).

Over the past decade, research focus on the health integration of immigrants in Norway has increased (Næss, 2019), exploring the differential health outcomes and health care utilization patterns of immigrants. Most of the available research on migrant health in Norway focuses on problems of immigrants’ access to and their utilization of the health care system in general (Mbanya et al., 2019; Næss, 2019). Research addressing the niche of migrant maternal health has mainly concluded that cultural diversity poses a challenge for the current Norwegian maternity care model (Lyberg et al., 2012). Other Norwegian studies on migrant maternal health address topics such as: nutrition (Garnweidner et al., 2013); domestic violence (Garnweidner-Holme et al., 2017); post-natal feeding practices (Wandel et al., 2016); risk of stillbirth (Vik et al. (2019); and migrant women’s experiences and perceptions of services (Egge et al., 2018; Glavin & Sæteren, 2016; Herrero-Arias et al., 2020; Viken et al., 2015). This study adds to the body of research by exploring how immigrant women experience and navigate the Norwegian Maternal Health Service during pregnancy and childbirth.

## The maternity health system in Norway

Norway’s social policy is founded on principles of universalism which require that everyone in society has equal entitlement to receive social goods, such as health and education. One of the definitions of universalism identifies the absence of means testing or differentiating between rights and needs (Rothstein, 1998). According to this definition, services therefore are available to all, but they are available in a standardized and uniform way, “embracing the entire citizenry … without the application of economic needs-testing” (Rothstein, 1998, p. 20). The standardized nature of universal welfare provisions may seem rigid because they can be less adaptable to individual desires and demands in comparison to more privatized systems (Rothstein, 1998). More interested in the links between government and its citizens through universal policy, Rothstein further makes the links between institutional trust and the generation of social capital (Rothstein & Stolle, 2003), and the role played by “street-level bureaucrats” as both impartial and conduits of social trust (Rothstein & Stolle, 2001) through what he terms “ethical universalism” as a mechanism for a fairer and more just society (Uslaner & Rothstein, 2016).

The general structure and scope of maternal health services and delivery is universal across Norway, meaning that all pregnant women regardless of legal residency status are entitled to equal maternal health services. The Norwegian Maternal Health Service (hereafter: NMHS) in this study refers to the health services provided to a woman during pregnancy and childbirth by a doctor, midwife, and/or nurse at a community health centre (*Helsestasjon* in Norwegian) or hospital. Maternal health care in Norway is offered at two organizational and professional levels; “specialized doctors at hospitals and general practitioners in municipalities perform maternity care services in cooperation with midwives on both care levels” (Gamst, 2012, p. 3). Although historically, general practitioners (GPs) were primary maternal health care providers, reforms beginning in the 1970s gradually shifted this responsibility to midwives to improve birth outcomes, and “from 1995 municipalities were bound by law to offer midwifery services” (Gamst, 2012, p. 4).

Midwives in Norway are specialized nurses (Lukasse et al., 2017). Today, maternal health services are mainly delivered by midwives at community health centres. A combination of antenatal consultations with a midwife and a GP is recommended in national clinical guidelines (Synne et al., 2005); however, women can choose who they want as their maternal health care provider. Midwives at the community health centre carry out most antenatal check-ups except ultrasounds, which are done at a hospital (Lukasse et al., 2017). Pregnant women are either referred to their community health centre by their primary care physician or they contact the centres directly to register themselves and make an appointment with a midwife. If the pregnancy is normal, the women continue to see their midwives. If the pregnancy is high risk, or if complications occur, the woman is referred to a gynaecologist or other relevant specialists for additional surveillance. Most births take place in hospitals in Norway with very few home births (Lukasse et al., 2017) and midwives at obstetric hospital wards have a prominent role in births, unless the pregnancies and subsequent births are deemed high risk. Women can give birth in the hospital with or without non-medical pain relief options, water births, and hotel stays after birth if the birth is not high risk. In the case that there is risk, pregnant women are taken to medically equipped obstetric wards for assisted birth and caesareans. According to Dahlberg et al. (2016), the midwives have an important role in helping women towards a normal birth, promoting the women’s own capacity to handle the birth, and to have a positive birth experience.

## Methods

A qualitative study design was adopted to gain a detailed understanding of immigrant mothers’ experiences and reflections. Following ethics approval from the Norwegian Social Science Data Services (NSD-234675), purposive sampling was used to recruit eleven immigrant women. The interviewees were recruited from public kindergartens and community health centres in two Norwegian municipalities with a high population of people with immigrant backgrounds.

### Overview of participants

The eventual sample comprised women from nine different ethnic and cultural backgrounds who had carried out their pregnancy and birth in Norway. The women had different reasons for immigration to Norway, length of residence in Norway, education level, and language proficiency in Norwegian and English. All interviewees were university educated and communicative in English despite being from different countries. This sample is therefore not representative of the most vulnerable population of immigrant women in Norway.

### Data collection

Once written and verbal consent to participate were obtained from the immigrant mothers, semi-structured interviews were conducted by the first author between October 2019 and March 2020, in person, by telephone, and online via Skype. The in-person interviews were conducted at the place of the interviewee’s liking, which was mostly in public kindergartens with their attending children, in their homes, or in cafes. The interviews ranged from thirty minutes to two hours in length and were carried out in three languages—nine in English and two in the participants’ own languages, Farsi and Kurdish, in which the first author is fully proficient. Biographic details were collected before the immigrant women were asked to recount their experiences of navigating and negotiating the NMHS during their pregnancies and births. Despite their diversity, there was a notable commonality in their experiences.

Participants were recruited by contingent purposive sampling (Hood, 2007), whereby the initial criteria for sampling were reiterated in response to challenges of accessibility to this participant group. As a result, homogenizing criteria such as the immigrant women’s period of residency in Norway by the time of their first pregnancy and birth, background and reason for immigration were compromised in favour of a more diverse and richer data set highlighting experiences of meeting and navigating the NMHS. The sample size was determined by both logistic factors binding the research period, and also the quality of the data.

### Data processing

The interviews were audio-recorded and transcribed verbatim by the first author. To protect the participants’ confidentiality, all were given pseudonyms, and identifying details were anonymized after transcription. The data were analysed using thematic analysis (Braun & Clarke, 2006). The interviews followed a semi-structured questioning format; therefore, the resultant data were first organized into general categories of the birth and pregnancy experience, and expectations of the health service. The eventual themes were inductively derived from carefully reading the interview transcripts several times and writing memos on what they expressed as their most significant experiences. In order to ensure rigour, the emergent themes were collaboratively assessed by all authors before reviewing and conceptualizing them against the literature. The themes identified were approach to pregnancy, monitoring and follow-ups, approach to birth and communication during labour.

### Data

The themes reflected the issues identified in these interviews which related to the women’s expectations of the services they should have and did receive, comments on whether these expectations were met, and if not, why. The women reflected on the differences and similarities to their home countries and how they navigated this new system. Some did this successfully, other less so. Communication between the women and the health professionals was identified as very important. These reflections covered the antenatal care and monitoring provided during their pregnancies, and their experiences immediately preceding and during birth.

## Findings

The community health centres were the first contact with the Norwegian health care system for many of the immigrant women interviewed, at which point most of them had little knowledge about the Norwegian health care and maternity care system. Furthermore, many of these women had not yet developed sufficient Norwegian language skills, were not in the workforce, and had not established a social network in Norway. All the mothers gave birth in hospital, with most giving birth within the first two years of their immigration to Norway. Their experiences on the navigation and negotiation of the NMHS are presented in this section under the themes identified in the methods.

### Approach to pregnancy

Some women felt that the approach to pregnancy in Norway was empowering. Zara from India was surprised that she was not asked to change her lifestyle during pregnancy. She was instead encouraged to continue with her regular work activities and to exercise, which enabled her to maintain her independence throughout her pregnancy. She would not have been able to enjoy the same freedom if she were pregnant in India because of different cultural expectations about how pregnant women within her social group should behave. She said:

Yeah, it is totally different [here]. In India, I would say there is more pampering. Here, it’s, ‘do whatever you like and keep doing what you did’. I am like an independent woman and like this [Norwegian] system very much than that one [in India]. Because there they would say, ‘oh, you’re pregnant, do not do this or that,’ haha. But here, if you like jogging, you can jog, you can play, you can swim, you’re totally free … Here, you’re free as a bird!

The Indian mother appreciated the freedom permitted in Norway, where a normal pregnancy is not considered as a particular risk to women or treated as a medical condition.

Another mother, Samantha from Serbia, also commented on the difference in birth perception in her home country compared to Norway. She was surprised by the different services available during the birth process, and noted the calmness of the birth environment, stating:

Everything was good. The midwives … they told me I could relax, I could take a bath … in my country, when you go into labor, there is no eating until you give birth. But here, you get dinner … It was a very good experience.

The Indian and Serbian mothers both focused on some differences in the approach to birth from their home countries which they considered positive and liberating.

### Monitoring and follow-ups

One aspect of the NMHS several interviewees found worrying and frustrating was the perceived lack of monitoring and follow-up during pregnancy. The relatively natural and hands-off approach to maternity care was criticized. Although most of these women did not have high risk pregnancies, they felt that this approach made them anxious and insecure.

Patricia from Indonesia compared NMHS with the services provided in Indonesia, where ultrasounds, for example, are undertaken monthly for paying patients, whereas in Norway, typically one ultrasound is taken between weeks 17–19 (The Knowledge Centre for Health Services, 2008). She was comparing two very different systems, reflective of differing policy frameworks and representing different user characteristics in willingness and ability to pay for private services. Nevertheless, the difference made her nervous.

You get curious. ‘Is the baby okay? Is it no okay?’, you know, just waiting for that first ultrasound [in Norway] seems like a very, very long time. You really want to know [how the pregnancy is going].

Cindy, a Chinese mother, had her first baby in her home country and her second in Norway. She explained her dissatisfaction with the monitoring and follow-ups by comparing the two broader welfare systems. She suggested that the Norwegian system of minimal antenatal scans may be explained by the welfare system:

In Norway they think, it doesn’t matter if you have a normal healthy baby or not, because post-birth support is available. Whereas in China, if something is wrong with the baby, the government doesn’t have the benefit to support you.

In her opinion, Norway, as a wealthy country with generous universal welfare provisions, could support families and their children if they had special needs; therefore, she posited that the NMHS did not prioritize detecting risks as extensively or as early in pregnancy as in China, whose welfare system relies on the assumptions of collectivist and familial provisions.

Merve from Germany experienced multiple late-term miscarriages in Norway. She believed they could have been prevented if the NMHS and maternal health care providers had observed her more closely during pregnancy. Believing her two miscarriages were caused by infrequent antenatal check-ups and being told there was nothing more that could be done except, “*you have high blood pressure, there is nothing we can do for you. So, wait at home. We will look again*”, she decided for her next pregnancy to have her baby in Germany, where she birthed a healthy child. She noted:

The medical [advice] they gave here [Norway] was different. The medicine that I was given here, I showed to my gynecologist in Germany, and she said no pregnant woman can get this. We stopped this in Germany 30 years ago. So … I understand that in this way Norway is behind 30 years maybe …

This mother criticized the Norwegian approach to pregnancy as laidback, and she was dissatisfied with her experiences. She did not trust the NMHS and its care providers’ competence in dealing with high-risk pregnancies. She found the uniform treatment and emphasis on the normality of pregnancy and birth as reasons to blame for her late-term miscarriages. Her distrust of the NMHS was justified when she moved back to Germany for closer antenatal monitoring during her third pregnancy and gave birth to a healthy baby.

Zara from India explained how she handled uncertainty during pregnancy. She consulted her sister-in-law, who is a doctor in India, and stated that “*she forced me and my husband to take a scan, like ultrasound, and confirm everything is okay, whether the baby has formed in a proper way*”. Given that the Norwegian system typically provides one ultrasound in the second trimester (weeks 17–19), Zara went to a private clinic in Norway and paid for the scan at 8 weeks. She continued:

I had very heavy vomiting, so I consulted the doctor and asked for tablets, but here they said no tablets … so I got the medicine from my sister in-law in India to subside the vomiting. That was helpful.

This mother’s experience illustrates her proactivity in bridging the gap between her expectations of maternal health care, which was not only shaped but continually influenced by medical practice and advice from her social network in her home country.

### Approach to birth

Birth was described as a challenging event by all the mothers. The two main contributing issues were linked to the degree of medical intervention and communication with health care professionals. These are presented in order. Excerpts from the interviews illustrate how discrepancies between the women’s ideal birth plans and the standard Norwegian birth approach were managed.

The degree of birth medicalization in the NMHS posed dilemmas for some mothers in their birth plans. Many of the participants came from cultures where they described birth by C-section as a more common elective option in private health care settings compared to Norway. Although some of the mothers expressed a desire for more medical intervention during their pregnancy and labour, some acknowledged a developing confidence in the Norwegian system. Consultations with midwives contributed to their acceptance about their body’s capability in the birth process.

Melika from Iran illustrates this growing confidence. At first, she wanted a highly medicalized birth, stating it was important for her to be able to “*see monitors and wires*” to put her mind “*at ease*”. But she formed a trusting relationship with her midwife, who gained her confidence in the NMHS and encouraged her to consider a natural birth.

I had researched caesarean birth, but I can say that it was my [Middle Eastern] midwife’s reassurance about my ability to give birth naturally because I had wide hips etcetera, that convinced me to go through with it. So that’s why I decided to forget about caesarean. Of course, this was against my mom’s advice, because she believed that I was physically too weak to push a baby out - and she was right, I have never been athletic or strong - but because research has shown that natural birth is better for the baby and the mom, I decided to accept the risk and do it.

When her birth was delayed, she was told that baby’s head had grown too big and that natural birth may be too difficult, but she insisted on going through with it, saying:

I have prepared myself for natural birth and will only do an operation if I can’t handle it. The hospital contacted me to ask what I wanted to do, and I explained that I wanted to go through with natural birth and only have a caesarean birth if there were complications.

Melika perceived pregnancy and birth as a risky period that needed constant monitoring. Consequently, she thought of technology as a safe and reassuring way of handling risk during childbirth, which would give her a sense of security. Her shift in mindset was facilitated by trust in her health care provider who had a similar cultural background as her but was working in the NMHS, as well as her own research into the topic.

Another mother, Parisa from Iran, also faced conflicting expectations between having a heavily medicalized birth and encouragement to have a natural birth. She felt pressured by the standardized Norwegian approach to birth, and doubted her ability to give birth naturally, while the emphasis on a natural birth in this system made her doubt its competency in performing caesareans and managing complicated births. Her pregnancy experience caused alarm as there was the possibility of a breech birth, which at the end did not occur. This enabled her to regain some confidence in the NMHS, stating: “*they have experience with all sorts of complications during natural birth, and so they are confident that even if the baby is breech, they will do a better and safer job birthing the baby rather than doing a C-section. So, their confidence is quite high in this regard*”. Her perceptions of the NMHS were influenced by her friends’ negative experiences with complicated births in Norway. Parisa would have liked the opportunity to choose her birth plan rather than be convinced to trust the natural birth process. Parisa interpreted the Norwegian model as less medicalized and as such more prone to risk, yet was convinced that because of the emphasis on this more natural approach, care providers would be more skilled in managing complications than performing a more medical birth. Hence, she compromised her wish for a medicalized birth with more trust in the capability of professionals.

### Communication during labour

Communication rather than language, played an important role in shaping the women’s experiences during labour and birth. Several of the mothers experienced poor communication from their health care providers.

Natasha from Israel had a difficult pregnancy and gave birth prematurely. When she went to the hospital, she was given medication to stop the labour; however, this was ineffective. She went into labour at 24 weeks, which is considered an extreme preterm birth that would require lifesaving support (Syltern et al., 2018). She stated that her care providers at the hospital were making plans about her baby’s resuscitation before the birth without involving her in the process.

I wanted to keep the baby inside. They [maternal health care providers] had something else in their mind … I didn’t know they were preparing me to give birth for two days until I was transferred to give birth … I even let my husband leave the hospital not knowing I was going to be induced soon. He didn’t know what was happening either, even though he is Norwegian.

‘Where [are] you take[ing] me? Where is the doctor?’ That’s what I was saying to the nurses. ‘Don’t worry … you just need to relax’ [the nurses said] … They didn’t tell me that I was going to give birth we didn’t know what was happening … Oh, now I’m emotional … that was hard.

Implicit in this Israeli mother’s description of the birth is the lack of meaningful communication. She felt that she was not informed about decisions that were made about her and that she was treated as a passive participant. What is experienced as inadequate communication here is possibly related to the perceived locus of expertise, which resides with the medical practitioners, not the mother, father, or other patients. This is a consequence of increased medicalization of the pregnancy and birth process. This experience contrasts with the perceived under-medicalization of the NMHS by the other interviewees.

Shania from Canada also experienced challenges with communication during labour. She was surprised that she would not have one constant midwife who would follow her throughout pregnancy and birth, as was common in her country. She said, “*it’s a nice idea to think you have one person who follows you before the pregnancy, is there for your birth and follows you after*”. She assumed that the communication between the woman giving birth and midwives would be better if they had already established a relation before the birth, as had Melika, the Iranian mother, and that the midwife at birth would be better acquainted with the patient. In Norway, several different midwives may be involved during a woman’s pregnancy and birth. One of Shania’s aims for her labour was to manage without epidural pain relief. She found that because she had not asked for it at the beginning of her labour, it was not provided to her when she eventually asked for it. She reflects: “*I would advise any future women giving birth in Norway who are apprehensive, if they want an epidural, they better ask for it right away!*” She identified part of the issue as the poor interaction and communication between her and the changing midwives during her labour about the labour process.

## Discussion

The women’s narratives raise several issues. The expectations the women had of their interactions with the professionals and the provisions of the health services were largely influenced by their knowledge of the systems and practices in their home countries. The interviewees in this study came from different countries and had diverse cultural backgrounds. They brought with them knowledge and ideal expectations of the types of care they wanted to receive during maternity which were shaped by their backgrounds and sociocultural positions. All the women had little knowledge of the NMHS. They were also coming into a system shaped by the principles of universalism which differed considerably from the guiding principles for the provision of health care in their own countries. For some women, having additional resources such as material wealth as well as professional and family networks that could be marshalled made a difference to their experiences. These were used to either enhance or bypass the Norwegian provisions. Some of the women experienced significant tension related to their expectations of highly medically supported or directed pregnancies and births. While the medical profession is central to maternity care in Norway, it is also expected that birth should be as natural as possible. The idea of pregnancy and birth as carrying unacceptable risk without medical intervention was paramount for some women who sought certainty and control. For some, this influenced the extent of their satisfaction with the Norwegian health system. Therefore, how these women experienced the Norwegian maternity care varied. While some were able to compensate for the discrepancies between what they perceived to be on offer of the NMHS and their expectations of maternity care, others had to adapt. The differences in the Norwegian approach to maternity care subsequently caused positive responses as well as anxiety, frustrations, and even changes in these women’s perceptions of maternity care. These experiences are analysed as consequences of medicalization, membership in and use of social networks, their participation in decision making and what implications these have for Norwegian maternity care in its universalist model. These discussions are presented in order.

### Experiences of under-medicalization

Peter Conrad (2013) defines medicalization as “the process by which former nonmedical problems become defined and treated as medical problems, usually as diseases or disorders” (p. 196). Many countries’ maternity health care systems have become increasingly medicalized, affecting how pregnancy and birth are treated. Advances in biomedicine and technology changed pregnancy and childbirth significantly during the 20^th^ century, creating a shift from the home to the hospital (Riessman, 1983), taking pregnancy and birth from a natural process to a medical event (Prosen & Krajnc, 2013). These advances allowed for closer surveillance and control of pregnancy and birth (Prosen & Krajnc, 2013), and significantly decreased mortality rates for mother and baby. The medicalization of maternity today entails antenatal screenings, the use of epidurals, birth by caesarean and the like. Consequently, pregnancy and birth have been reconceptualized as a result of increased medicalization from a “natural, normal, woman-centered event” to “a dangerous time wherein a woman and her fetus are at risk and in need of constant medical monitoring and intervention” (Parry, 2008, p. 785).

In this study, some participants such as Parisa expressed the push towards the more natural and midwifery-oriented model of birth in Norway as disconcerting. There is evidently a tension between the medical and more natural model of birth which has been debated among researchers in this field.

Unlike the medical model, the midwifery model [here, the more ‘natural’ model] consistently sees the needs of the mother and the fetus as being in harmony, the two as one “organic unit” and posits that both pregnancy and childbirth are “health and entirely normal condition[s]” (Simonds et al., as cited in Brubaker & Dillaway, 2009, p. 37).

This model of maternity care is “woman centered and holistic” (Brubaker & Dillaway, 2009, p. 37), meaning that the midwives try not to intervene, and rather attempt to empower the mother to take control of the pregnancy. In contrast, Brubaker and Dillaway (2009) state that the natural birth approach denies women choice and agency, “essentializes” women’s childbirth experiences, and reflects class and race bias. Others argue that birth medicalization empowers women by giving them control over their maternity care (Prosen & Krajnc, 2013), “accompanied by an implicit promise that risks can be managed” (Hall et al., 2012); whereas “second-wave feminists have viewed the medicalization of childbirth as medical authority’s usurpation of authority, choice, and control over women’s reproduction” (Brubaker & Dillaway, 2009, p. 35). The participants of this study who criticized the under-medicalization of maternity care in Norway would have felt more empowered if they had the choice of having more medicalized care.

Additionally, the participants expected more frequent surveillance to control for and perhaps respond to possible abnormalities in the foetus early in the pregnancy, as the Chinese and the Indian mothers pointed out. Cindy compared the limited antenatal care in Norway to the rigorous one in China, emphasizing that detecting abnormalities during pregnancy was a priority in China because of the serious implications it would have for the family in the absence of state support. The German mother, who had experienced several miscarriages in Norway, was particularly dissatisfied with the quality of maternity care in Norway, and care she received in Germany reinforced her view that the NMHS was not only under-medicalized, but also outdated. The medicalization of maternity care is therefore a wider reflection of the state and its policies on family and health care. As such, the medicalization of pregnancy and birth varies across different maternity care models. For example, “whilst the UK and USA are highly medicalized, Scandinavian countries and the Netherlands are less so, and there, where birth is seen as a normal physiological process, rates of clinical interventions such as Caesarean sections are significantly lower” (Nettleton, 2013, p. 144). On the critique of birth medicalization, Stoll and Hall (2013) state, “the medicalization of birth is a cultural expression of the core values of technocracy. In a technocratic society, a highly functional natural process, like birth, is viewed as dysfunctional and in need of technological intervention” (p. 1501), and in a “risk society” (Beck, 1992) the services are organized to handle birth as a risk. Benyamini et al. (2017), found that “the feeling of fear could lead to an appreciation of birth medicalization”. Although most births are normal, “they are treated like an illness, and mothers as patients” (Nettleton, 2013, p. 143). The context in which childbirth occurs may therefore influence the experience of the pregnancy and birth (Macpherson et al., 2016), and to what degree it is medicalized.

### Social networks

The data show that the interviewees’ social networks might have contributed to how they encountered the NMHS. They had friends who informed them about what to expect from the NMHS, which can be considered system knowledge, an important type of knowledge for navigating the health care system (Willis et al., 2016). Willis et al. (2016) define system knowledge as “a form of knowledge applied to the navigation of the field of healthcare” (p. 210). System knowledge is necessary for “effective decision-making as patients navigate their way through the healthcare system” (Willis et al., 2016, p. 204). This can either be acquired from experience or assumed from “networks of privilege” (Willis et al., 2016, p. 202), like social networks, to gain an advantageous understanding of the system. The latter was the case for some of the women interviewed who relied on their social networks to inform their decisions about their pregnancy and birth experiences in Norway.

Another example of assumed system knowledge evident in the data was linked to a midwife who acted as a cultural bridge builder (Næss, 2019) in acquainting some mothers with the NMHS and helping them navigate and develop trust in it. This midwife was able to gain the trust of Melika, an Iranian woman who was doubtful that the under-medicalization of maternity care would meet her needs. But through the relationship, she was encouraged to believe that she was capable of giving birth naturally. This midwife was a network of privilege for Melika in gaining system knowledge about the NMHS. This midwife simultaneously acted as an agent of universalism who gained the confidence of a sceptical immigrant woman in the NMHS.

Other women had transnational ties that helped them compensate for the differences they encountered in the medicalization of antenatal care. This, we argue, is a form of transnational system knowledge used for bridging perceived gaps in a new medical model. One of the interviewees, for example, had a sister-in-law who was a physician in India who told her to get an earlier ultrasound and sent her medications the interviewee could not obtain from her Norwegian physician.

System knowledge as a concept is important in the immigration setting because immigrants must transfer, adapt, and sometime relearn this knowledge when encountering a new health care system. Social and cultural capital, such as networks, education, and communication skills, are therefore important assets in the transitioning of system knowledge which can enable the navigation and even the negotiation of a new health care system. The central point is that the interviewees’ social networks, their social capital, and experiences contributed to how they applied system knowledge to navigating and negotiating the NMHS; hence shaping how they experienced and interpreted it.

### Participation in decision making

Research shows that the relationship between health care provider and patient plays an important part in generating satisfaction with childbirth (Benyamini et al., 2017; Clesse et al., 2018; Hildingsson et al., 2021), but for immigrant women, this relationship may be influenced and disturbed by several factors. Immigrant women’s abilities and styles of communication vary because “culture and ethnicity can influence significantly how people communicate their healthcare needs” (Shrestha-Ranjit et al., 2020, p. 1698).

The Israeli mother who had a premature birth felt that she had no opportunity to participate actively in decisions around her labour, and she experienced the event as disempowering. What she experienced could be considered as a form of over-medicalization over which she had no control. She was not involved in what, for her, was a critical life event. Although the acuteness of her condition may have led the health care professionals to prioritize intervention over communication, she felt that her involvement in decisions made about her body and her infant was necessary.

### Norwegian maternity care model and the universalist state

A common experience for most interviewees in this study was their expectation of a more medicalized service than the NMHS offered. For some of the women, their desires for more medical intervention led them to partially deviate from the universally provided services, seeking services outside the NMHS through private health institutions in Norway or abroad, including from their home countries. The women who were able to seek services outside the NMHS were those with financial resources as well as connections and knowledge about alternative services. Similar to Benyamini et al. (2017), we found that women’s attitudes towards medicalized birth varies, and that this is often related to their sociodemographic and sociocultural background. What differs is that women in this study requested and expected a more medicalized maternity care than was offered, while women in the study of Benyamini et al. (2017), who were also used to an advanced medical system, were more reluctant to accept medicalized birth.

The medicalization of childbirth and pregnancy, however, is one of degree. The Norwegian system has, as much of the western world, embraced a medical framework for the management of pregnancy and birth, albeit not to the degree some of the women desired. This is because a more natural approach to maternity care with fewer unnecessary medical interventions has been emphasized in Norway since the 1970s midwifery reforms, both for better birth outcomes and more positive birth experiences (Dahlberg et al., 2016; Gamst, 2012). The Norwegian maternity health care system is continuously changing with the development of medical knowledge and technology, as are the standards of practice in maternity care. The use of caesarean births and epidurals are steadily increasing in Norway (Dahlberg et al., 2016), and recently, politicians have implemented earlier ultrasounds screenings during pregnancy.

As pointed out by Rothstein (1998), a universal health care or service system limits individual choice and instead directs individuals into a standardized and conforming system. Such systems may be experienced as rigid and less adapted to individual demands, and difficult to adjust to, especially for those whose experiences lead them to believe this system exposes them to risk. But Rothstein’s interpretations of universalism are also concerned with the generation of social trust and social capital to achieve more just and fair systems. Here, the women from diverse countries experienced a very different health system from that which they were used to and, being distrustful, they circumvented it using individual choice. Others, however, found it liberating, and in this way, their experiences generated social trust and their own social capital which can contribute to greater social cohesion (Fonseca et al., 2019). These women are not representative of all immigrant women seeking maternity services in Norway, but in order to achieve the type of social trust Rothstein conceptualizes for a just and fair system, a universalist system needs to find ways to overcome the kind of social distrust some of the women in this study display. While lesser educated women with fewer resources may not have the wealth of social networks available to these women, they will almost certainly have some apprehensions about different systems, especially if communication forms a barrier to expressing desires and needs. Greater medicalization of childbirth need not only apply to resource-rich women, but expectations of greater care through medical attention may still be present in many immigrant women. A universalist system which seeks to engender social trust must pay attention to difference rather than sameness and seek to overcome concerns through generating genuine trust in the systems.

Some of the interviewees were satisfied with the system and some changed how they perceived it. The Middle Eastern midwife could be interpreted as an agent for the universalism of the Norwegian welfare state by working as a street-level bureaucrat (Lipsky, 1980) to make the NMHS more accessible and acceptable to those who come from different countries. This act of cultural translation and gaining of support from non-Norwegians for the NMHS is important in sustaining the universalist welfare state.

## Conclusion

Expectations and demands differ, and they differ with situations. They may be related to culture, social class, social networks, knowledge, and personal experiences. While these women do not represent any group, ethnicity, or culture other than their own, the Norwegian maternity care model differed from their expectations. Some had generally positive experiences, whilst for others, their experiences fell short of their expectations. Although this study was influenced by limitations such as access, time, and language, the findings of this study contribute to understanding the implications of universal maternal health care through the perspective of immigrant service users. This article underscores the issues of diversity and choice within a universal system, contributing to a larger ongoing discussion on universalism in the Nordic welfare system.
